# Bacteroidales Secreted Antimicrobial Protein-1 receptor recognition is coupled to protease-activation to form bactericidal pores

**DOI:** 10.64898/2026.08.05.742979

**Published:** 2026-08-05

**Authors:** S N Mostyn, K Flores, G Hedger, H Nagaraj, S L Rouse, L E Comstock, D Bubeck

## Abstract

Bacteroidales secreted antimicrobial proteins (BSAPs) are diffusible MACPF-domain toxins that mediate intra-species antagonism in the gut microbiota. Here we define the mechanism of action of BSAP-1 from *Bacteroides fragilis* , showing how target specificity encoded within the N- and C-terminal domains is coordinated with pore-forming activity of the MACPF. We show that specificity of the toxin for its receptor is mediated by an extended interface comprised of the BSAP-1 C-terminal domain and residues on the receptor that differ from the orthologous protein of BSAP-1 producing strains. On the surface of susceptible cells, BSAP-1 undergoes proteolytic cleavage of an N-terminal regulatory domain, triggering its assembly into oligomeric pores. Cryo-electron microscopy of membrane-inserted BSAP-1 reveals a 13-subunit transmembrane β-barrel pore formed through canonical MACPF rearrangements. Comparative modelling supports a conserved oligomerization mechanism across the BSAP family despite diversification of receptor-binding domains that target either proteins or glycan receptors. Together, these findings establish BSAP-1 as a receptor-targeted, protease-activated antibacterial MACPF toxin and provide a framework for understanding how gut Bacteroidales spatially restrict toxin activation to shape strain-level competition.

## Full Text Availability

The license terms selected by the author(s) for this preprint version do not permit archiving in PMC. The full text is available from the preprint server.

